# Long-Term Implanted cOFM Probe Causes Minimal Tissue Reaction in the Brain

**DOI:** 10.1371/journal.pone.0090221

**Published:** 2014-03-12

**Authors:** Thomas Birngruber, Arijit Ghosh, Sonja Hochmeister, Martin Asslaber, Thomas Kroath, Thomas R. Pieber, Frank Sinner

**Affiliations:** 1 HEALTH – Institute of Biomedicine and Health Sciences, JOANNEUM RESEARCH, Graz, Austria; 2 Division of Endocrinology and Metabolism, Medical University of Graz, Graz, Austria; 3 Division of General Neurology, Medical University of Graz, Graz, Austria; 4 Institute of Pathology, Medical University of Graz, Graz, Austria; University of Nebraska Medical center, United States of America

## Abstract

This study investigated the histological tissue reaction to long-term implanted cerebral open flow microperfusion (cOFM) probes in the frontal lobe of the rat brain. Most probe-based cerebral fluid sampling techniques are limited in application time due to the formation of a glial scar that hinders substance exchange between brain tissue and the probe. A glial scar not only functions as a diffusion barrier but also alters metabolism and signaling in extracellular brain fluid. cOFM is a recently developed probe-based technique to continuously sample extracellular brain fluid with an intact blood-brain barrier. After probe implantation, a 2 week healing period is needed for blood-brain barrier reestablishment. Therefore, cOFM probes need to stay in place and functional for at least 15 days after implantation to ensure functionality. Probe design and probe materials are optimized to evoke minimal tissue reaction even after a long implantation period. Qualitative and quantitative histological tissue analysis revealed no continuous glial scar formation around the cOFM probe 30 days after implantation and only a minor tissue reaction regardless of perfusion of the probe.

## Introduction

Implantable microelectrodes, biosensors and sampling probes are used to investigate the metabolism and the chemical composition of the interstitial fluid in brain tissue. All of these devices critically depend on substance exchange with the surrounding tissue [1]. Histological studies have frequently reported a glial scar, a tissue reaction that surrounds long-term implanted probes. The dense nature of the scar tissue hampers substance transport and therefore the function of implanted probes [2]. Glial scar formation and biofouling on probe surfaces and interface membranes are major factors decreasing probe performance over time. Compared to biofouling, the glial scar has a 3–5 times higher impact on decreasing transport of small substances [3]. The precise mechanisms that influence the extent of tissue response to artificial implants are not completely understood [2], [4]–[7]. Though all invasive techniques cause implantation stress, perfusion probes like microdialysis (MD) or push-pull perfusion have additional stress factors caused by the chemical properties of the perfusate or shear forces due to perfusate flow [8], [9].

Cerebral open flow microperfusion (cOFM) is a relatively new sampling technique based on conventional open flow microperfusion [10]–[13] that allows sampling of large and lipophilic substances in brain interstitial fluid with an intact blood-brain barrier (BBB) to measure substance transport across the BBB. All materials used in the design of cOFM probes are chosen in order to minimize tissue reaction and glial scar formation. Compared to MD sampling, cOFM sampling is not based on a membrane and allows direct, unfiltered mixing of perfusate and interstitial brain fluid. Avoiding a membrane also minimizes adhesion of cells and substances to the probe's surface, avoids cell migration into a membrane, and reduces continuous irritation of surrounding tissue that is caused by the jagged MD membrane surface [14], [15]. The functional principle of cOFM is very similar to that of push-pull perfusion which was one of the first techniques developed to sample in brain tissue. A major drawback of push-pull perfusion is severe tissue damage around the probe [16], [17].

In the present study we aimed to evaluate the long-term effect of cOFM probe materials and design in regard to brain tissue reaction with a focus on day 15 after cOFM probe implantation, at which time BBB is reestablished [18]. We compared the histology of brain tissue around the cOFM probe with naïve frontal lobe tissue of the contralateral hemisphere and studied the effects of probe implantation and perfusion.

## Materials and Methods

### Animals

All animal protocols used in this study were approved by the Austrian Ministry of Science and Research (Ref.II/10b, Vienna). A total of 36 adult male Sprague Dawley rats (Harlan Laboratories, Udine, Italy) with a weight of 300–450 g were used in this study. Animals were allowed to acclimatize to the environment for at least one week after transportation before any surgical procedures were carried out. After probe implantation animals were housed individually in acrylic glass cages with a 12∶12 h light∶dark cycle, and food and water were available *ad libitum*. Appropriate animal care was provided by the staff at the animal care facility (Institute for Biomedical Research, Medical University of Graz, Austria).

### cOFM probe

**Figure 1:**
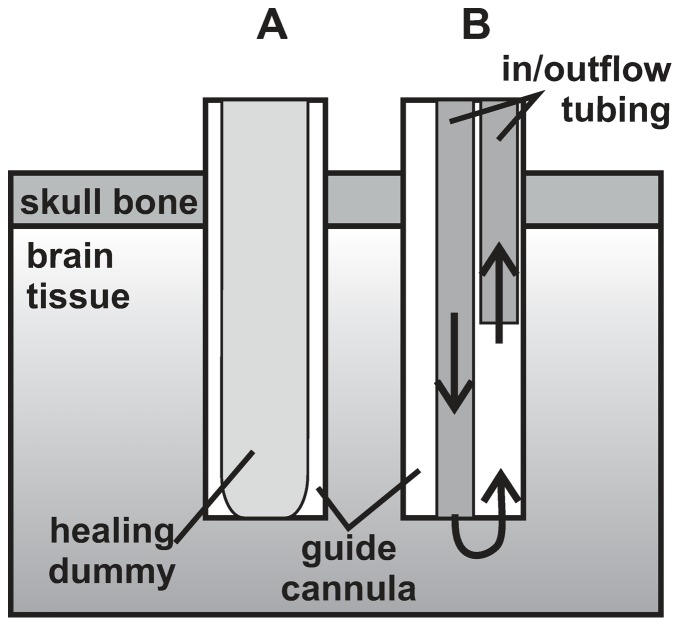
Schematic drawing of the cOFM probe tip during the healing period (A) and during sampling (B).

The cOFM probe (Fig. 1) consists of a 20 Ga fluorinated ethylene propylene (FEP) guide cannula that is inserted into the brain tissue and a healing dummy that provides mechanical stability during implantation. The healing dummy also prevents tissue ingrowth into the guide cannula during the healing period. The space between healing dummy and guide cannula that is needed for insertion and extraction of the dummy also allows the flexible guide cannula to follow the micro-motions of the brain tissue. Before sampling starts the healing dummy is replaced by inflow/outflow tubing.

### Implantation of cOFM probe

For cOFM probe implantation, the rats were anesthetized with a subcutaneous injection (0.015 ml/kg body weight) of a combination (2∶2∶1) of Fentanyl (0.05 mg/ml; Janssen-Cilag Pharma, Austria), Midazolam (5 mg/ml; Janssen-Cilag Pharma, Austria) and Domitor (0.1 mg/ml, Pfizer Corporation, Austria). The head was fixed in a stereotactic frame (KOPF Instruments, USA) and rats were prepared for surgery by shaving the head and disinfecting the skin with 70% ethanol. A spherical dental drill was used to drill a 1 mm hole into the skull leaving the dura intact. The dura was then punctured with fine forceps in order to create a defined opening of the meninges.

Using the stereotactic frame the cOFM probe was slowly inserted into the frontal lobe to a final position of 2 mm left from midline, 0 mm anterior to bregma and 1.5 mm below the dura. The probe was fixed to the skull bone using two anchor screws and biocompatible dental cement (iCEM Self Adhesive; Heraeus, Germany). All surgical procedures were completed within 30 min and anesthesia was terminated by subcutaneous injection of the anesthesia antagonists Anexate (0.1 mg/ml; Roche Austria GmbH) and Antisedan (0.5 mg/ml; Pfizer Corporation, Austria). For two days after surgery a daily dose of antibiotics Claforan (50 mg/kg, Sanofi-Aventis GmbH, Austria) and a pain treatment (Rimadyl; 0.1 mg Carprofen; Pfizer, Austria) were administered subcutaneously.

### Sampling

For cOFM sampling the rats were anesthetized with a minimal dose of Isoflurane (1%) for 3 hours. At first the healing dummy was replaced with inflow/outflow tubing and connected to two glass syringes (Hamilton, USA) placed in syringe pumps (Aladdin, World Precision Instruments, Germany). cOFM perfusate was pumped into the probe with a flow rate of 1 µl/min and samples were withdrawn at the same flow rate. Sampling was conducted for 2 hours. For two days after sampling a daily dose of antibiotics (Claforan, 50 mg/kg, Sanofi-Aventis GmbH, Austria) was administered subcutaneously.

In order to avoid chemical stress to the brain tissue the cOFM perfusate was composed to match brain interstitial fluid, modified from McNay et al. [19]. The perfusate was mixed under sterile conditions and consisted of 123 mM NaCl, 0.4 mM MgCl_2_ (purity ≥98%), 0.7 mM CaCl_2_ (purity ≥93%), 4.3 mM KCl, 1.3 mM NaH_2_PO_4_, 21 mM Na_2_HPO_4_, 4 mM glucose. All reagents were dissolved in sterile water (Aqua bidest, Fresenius Kabi, Austria). In order to remove possible bacterial contamination, the perfusate was filtered through a 0.22 µm sterile filter (Thermo Fisher Scientific, Germany). All reagents were purchased from Sigma Aldrich, Austria (purity ≥99%) unless stated otherwise.

### Experimental setup

A total of 36 rats were divided into 3 groups that differed in the duration of cOFM probe implantation: 3 days (n = 6), 15 days (n = 24) and 30 days (n = 6) between cOFM probe implantation and brain extraction. Each of the 3 groups was subdivided evenly in a perfused and a non-perfused group. Perfusion was carried out for two hours on day 1, 11 and 15 after cOFM probe implantation and sacrificed on day 3, day 15 and day 30, respectively, in order to characterize and compare the morphological changes between non-perfused and perfused animals. For euthanasia every rat was deeply anesthetized with a double dose of anesthetics as used for surgery. The animals were flushed transcardially with PBS to remove intravascular blood and avoid bleeding during cOFM probe extraction and brains were fixed with 4% paraformaldehyde. Extracted probes were checked for adherent tissue using scanning electron microscopy. After complete fixation, the tissue was embedded in paraffin and sectioned in coronal slices of 4 µm thickness. Slices were made serially throughout the cOFM probe implantation site.

### Scanning electron microscopy

Immediately after explantation, cOFM probes were pretreated according to standard protocol: Probes were fixed with 2.5% glutaraldehyde and 2% paraformaldehyde in 0.1 M cacodylate buffer for 1 h. Probes were then rinsed in cacodylate buffer, post-fixed for 1 h in 2% osmium tetroxide, rinsed again in cacodylate buffer and dehydrated in a graded series of ethanol. cOFM probes were critical-point dried in super-dry aceton, mounted on aluminum pins, sputter coated and examined under a scanning electron microscope (ZeissDSM 950).

### Implantation of microdialysis probe

As a positive control for effective staining comparable cerebral microdialysis (MD) probes (CMA 12, membrane OD 0.5 mm, cut-off 20,000 Dalton; CMA Microdialysis AB, Sweden) were implanted intraparenchymally in the frontal cortex of rats (n = 3) following the same procedure and stereotactic coordinates as described in section “Implantation of cOFM probe”. The animals were euthanized 15 days after MD probe implantation following the same procedure as in section “Experimental setup”. MD probes remained in the brain during fixation and sectioning.

### Immunohistochemistry and staining

Haematoxylin and eosin (H&E) staining was performed in at least 5 sections with 100 µm distance from each other following standard protocol. Two adjacent slides were selected for immunohistochemical Iba-1 and GFAP staining. Slides for Iba-1 were stained following standard protocol on a Leica BondTM Max autostainer (Leica Biosystems, Australia). Antigen retrieval was performed with ER1 for 10 min (solution provided by the manufacturer for epitope retrieval). Iba-1 antibody (Cat. No.: ab 15690, Abcam, Austria) was diluted to 1∶2,000 in Bond Primary Antibody (Cat. No.: AR9352, Leica, UK) and detected with BondTM Polymer Refine Detection (Cat. No.: DS9800, Leica, UK). For GFAP, slides were stained following standard protocol on a Ventana Benchmark ULTRA autostainer (Ventana Medical Systems, USA). Antigen retrieval was performed with CC1 for 30 min (solution provided by the manufacturer for epitope retrieval). GFAP antibody (Cat. No.: 258R-24, Cell Marque, USA) was diluted to 1∶500 and visualized with ultraView Universal DAB Detection Kit (Cat. No.: 760-500, Ventana Medical Systems, USA).

### Histological analysis

**Figure 2:**
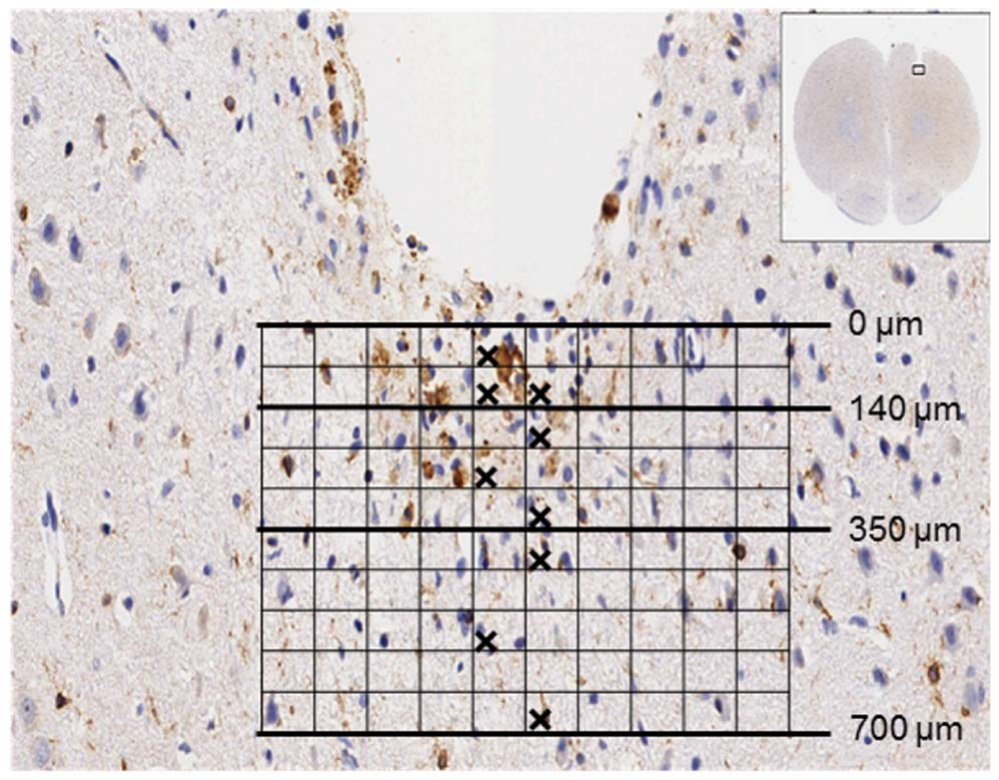
Schematic representation of quantification pattern. Each square has a size of 4,900 µm^2^; 200× magnification. Checked squares indicate the areas selected for cell count.

Brain sections stained with H&E were examined with an optical microscope in order to localize the probe implantation site. Qualitative assessment was carried out in all groups. Quantitative analysis of Iba-1 and GFAP positive cells was performed in the 15 days group only. Tissue around the probe was divided into three areas with different distances from the probe tip (0–140 µm, 140–350 µm, 350–700 µm) using the optical microscope (Axiocam, Nikon) at magnification 200× with an optical grid (Fig. 2). Two independent observers selected representative grid squares in the three areas and counted Iba-1 positive microglial cells and GFAP positive astrocytes and calculated the mean value in cells/mm^2^. In each section a comparable area on the contralateral hemisphere served as a control area with naïve tissue.

### Statistical analysis

Statistical analyses of the quantitative results of Iba-1 and GFAP staining were performed with R 2.13.1. A Friedman test for repeated measurement was performed to test for differences in the numbers of counted cells in four related groups (three different areas around the probe tip and the control area). This was done separately for perfused and non-perfused rats with a significance level set at 5%. Post hoc testing was done using two samples paired Wilcoxon Rank Sum tests to determine statistical differences between the number of counted cells in two paired groups (hemispheres in the same animal). The initial level of significance (5%) was adjusted by Bonferroni correction for multiple comparisons.

## Results

**Figure 3:**
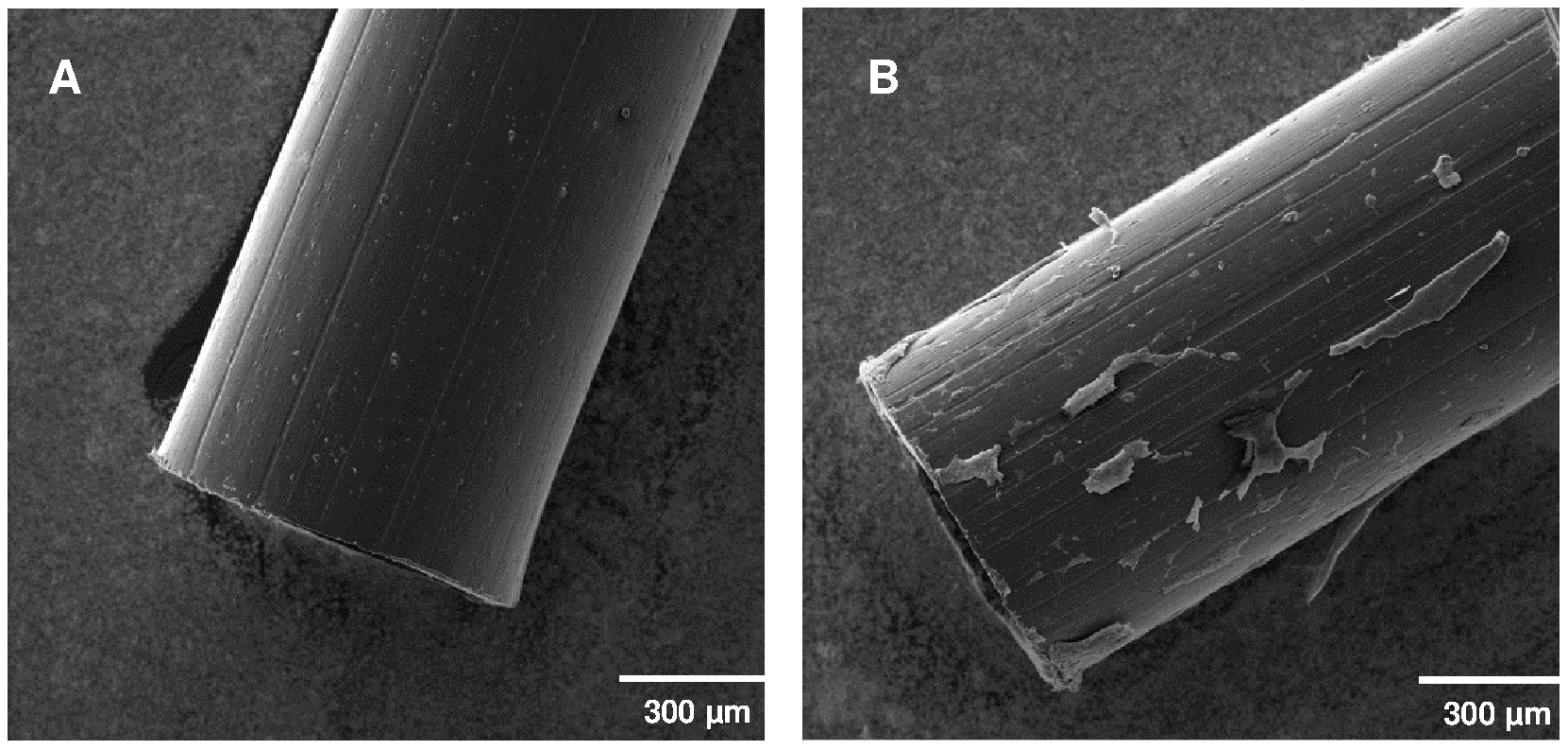
Representative scanning electron microscope image of non-implanted cOFM probe (A) and a cOFM probe implanted for 15 days (B).

For histological examination all cOFM probes were removed and carefully checked for adherent tissue using scanning electron microscopy. We found no tissue on any of the probes which would have caused underestimation of glial scarring in the remaining tissue section. Fig. 3 shows a representative scanning electron microscopy image of a non-implanted cOFM probe and a probe implanted for 15 days.

### H&E

**Figure 4:**
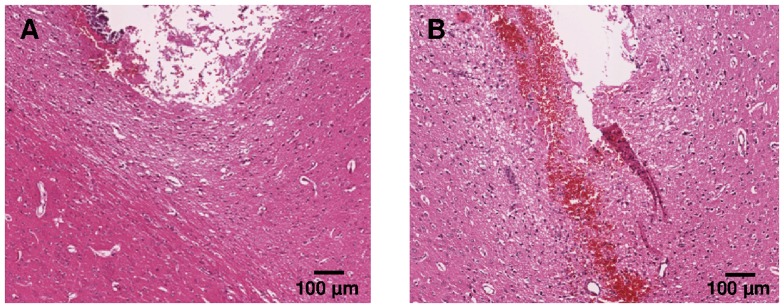
Representative H&E images of non-perfused (A) and perfused (B) rat brains 3 days after cOFM probe implantation.

**Figure 5:**
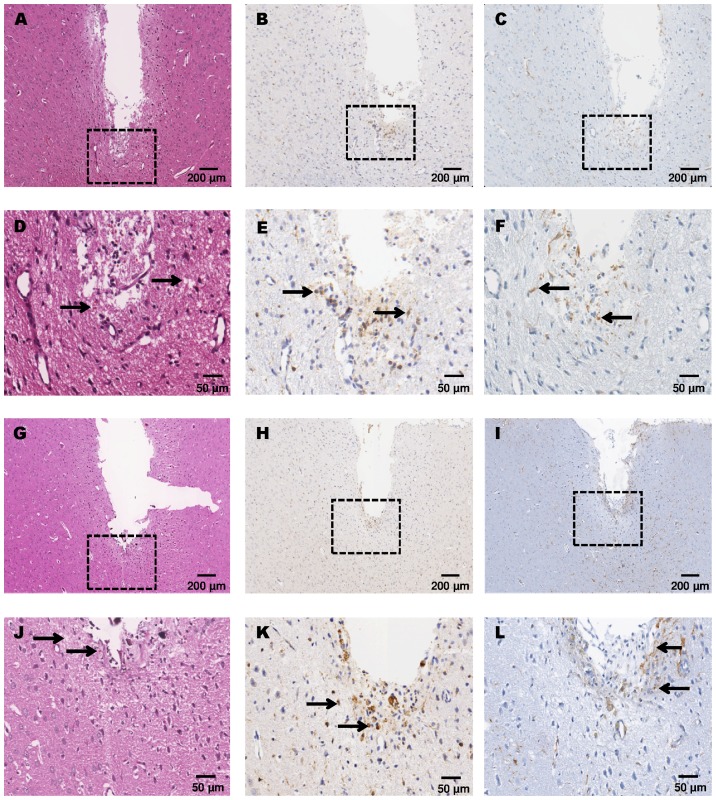
Microscopy of the cOFM probe implantation site in the frontal cortex after 15 days. Representative images of non-perfused (A–F) and perfused (G–L) rat brains. Adjacent brain slides were stained with H&E (A, D, G, J), Iba-1 for microglia (B, E, H, K) and GFAP for astrocytes (C, F, I, L). Rectangles with broken lines in A–C and G–I (50×) are shown at higher magnification (200×) in D–F and J–L, respectively. At this stage only a minimal residual edema is detectable with H&E staining in both non-perfused and perfused animals. Only a minor microglial (E, K) and astrocytic (F,L) reaction is visible directly adjacent to the cOFM probe implantation site.

Microscopic analysis of H&E stained samples on **day 3** after implantation showed residual erythrocytes at the tip of the probe indicating bleeding after probe implantation in both groups but less pronounced in the non-perfused group. Edema formation with minor tissue debris below the probe tip in implantation direction was detected only in close vicinity of the probe tip (Fig. 4). On **day 15** after probe implantation, residual blood and tissue debris had dissolved completely and minimal edema was detected (Fig. 5 A, D, G, J). On **day 30** after probe implantation, edema, residual blood, and tissue debris were completely regressed, hemosiderophages were detected occasionally.

**Figure 6:**
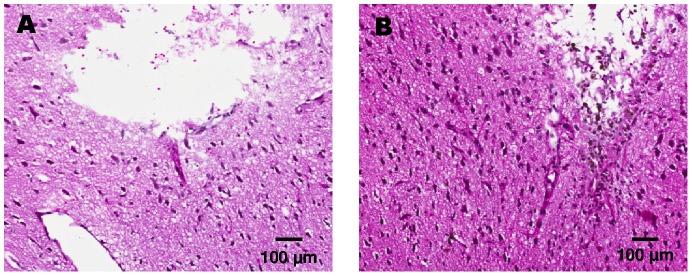
Representative H&E images of non-perfused (A) and perfused (B) rat brains 30 days after cOFM probe implantation.

None of the sections showed a continuous glial scar or pronounced tissue reaction in response to the probe (Fig. 6). No considerable differences were found between perfused and non-perfused groups.

### GFAP

**Figure 7:**
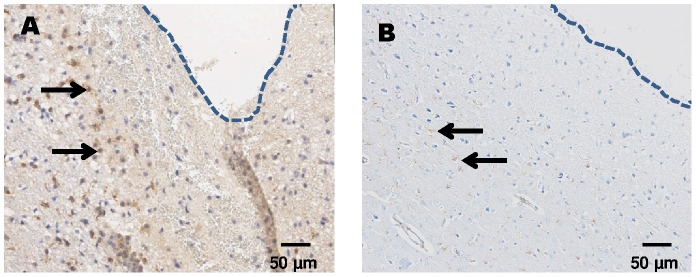
Microscopy of Iba-1 (A) and GFAP immunoreactivity (B) 3 days after cOFM probe implantation (200×).

**Figure 8:**
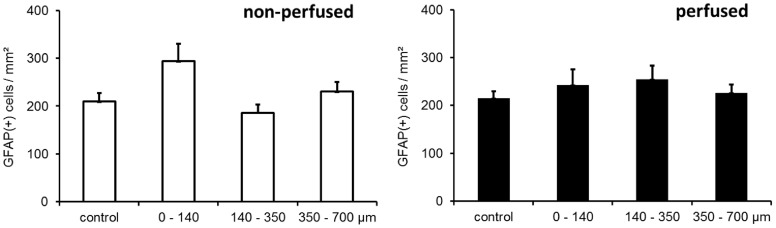
Quantification of GFAP positive cells 15 days after cOFM implantation at different distances from the probe tip and in the corresponding area on the contralateral hemisphere (control).

**Figure 9:**
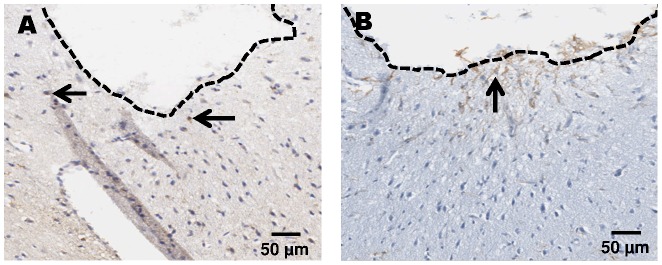
Microscopy of Iba-1 (A) and GFAP immunoreactivity (B) 30 days after cOFM probe implantation (200×).

**Figure 10:**
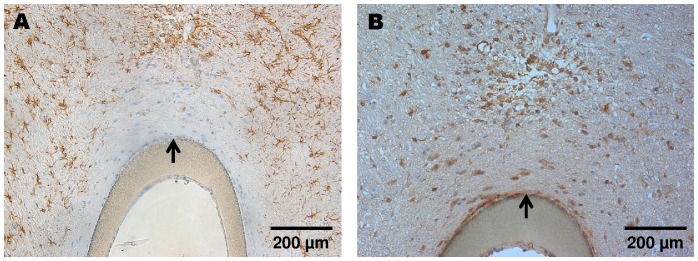
Representative images of the microdialysis membrane (CMA 12; cut-off 20,000 Da) implanted in the frontal cortex of Sprague Dawley rats. (A) Classic morphology of reactive astrocytes (GFAP) around the microdialysis probe 15 days after probe implantation. (B) Increased Iba-1+ microglia in close vicinity of the microdialysis probe 15 days after implantation. The arrows point towards the outer surface of the microdialysis membrane.

GFAP stains astroglia, the cell type mainly responsible for glial scar formation and probe encapsulation. On **day 3** after cOFM probe implantation, GFAP immunoreactivity was slightly elevated in both groups being more pronounced in the perfused group (Fig. 7B). On **day 15** after probe implantation, quantitative GFAP analysis was performed in the different areas surrounding the probe and in naïve control tissue from the contralateral side (Fig. 5 C, F, I, L). The quantitative analysis revealed no significant difference between the areas around the probe and the control area. Perfused and non-perfused groups showed no significant difference (Table 1, Fig. 8). On **day 30** after probe implantation, only a minor astrocytic reaction was observed in both groups (Fig. 9B). We did not observe a continuous astroglial scar in any animal. The implanted **microdialysis** probe showed a substantially increased astrocytic reaction around the probe and thus indicates an effective staining procedure (Fig. 10).

**Table 1:** Quantification of GFAP+ cells 15 days after cOFM probe implantation.

	non-perfused cells/mm^2^		perfused cells/mm^2^	
area	mean ± SEM	p value	mean ± SEM	p value
control	209±18	-	215±14	-
0–140 µm	294±36	0.067	243±33	0.687
140–350 µm	186±17	0.140	254±29	0.236
350–700 µm	230±20	0.553	226±17	0.774

p values were calculated by comparing GFAP cell counts in each area with the contralateral control area.

### Iba-1

**Figure 11:**
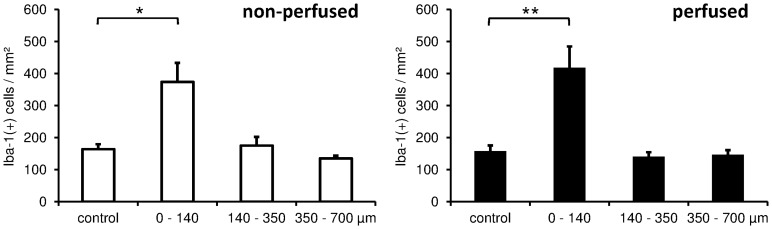
Quantification of Iba-1 positive cells 15 days after cOFM implantation at different distances from the probe tip and in the corresponding area on the contralateral hemisphere (control).

Iba-1 stainings show activated microglia after CNS injury. Microglia is the second most important cell type that indicates glial scar formation. On **day 3** after cOFM probe implantation, microglia activation was detected in the direct vicinity of the probe and was slightly more pronounced in perfused animals (Fig. 7A). On **day 15** after probe implantation, the quantitative analysis showed a significantly higher number of Iba-1 positive cells but only in close proximity (0–140 µm) of the probe tip. The number of Iba-1 positive cells in this area was significantly higher in both non-perfused (374±60, p≤0.05) and perfused (419±66, p≤0.01) brains compared to the control area in the contralateral hemisphere (Fig. 5 B, E, H, K). Areas more distant from the probe tip (>140 µm) showed no significant difference to the control area and no difference between perfused and non-perfused animals (Table 2, Fig. 11). On **day 30** after probe implantation only a minor Iba-1 immunoreactivity was observed at the implantation site (Fig. 9A). Similar to GFAP staining the implanted microdialysis probe showed a substantially increased microglial reaction around the probe and thus indicates an effective Iba-1 staining procedure (Fig. 10).

**Table 2:** Quantification of Iba-1+ cells 15 days after cOFM probe implantation.

	non-perfused cells/mm^2^		perfused cells/mm^2^	
area	mean ± SEM	p value	mean ± SEM	p value
control	164±16	-	158±18	-
0–140 µm	374±60	0.019	419±66	0.006
140–350 µm	175±27	0.837	141±13	0.390
350–700 µm	135±8	0.121	147±14	0.457

p values were calculated by comparing Iba-1+ cell counts in each area with the contralateral control area.

## Discussion

In this study we assessed the histopathological effects of long-term implanted cOFM probes in the frontal lobe with a special focus on day 15 when the blood-brain barrier is reestablished [18]. As a secondary aim we investigated the histopathological impact of cOFM probe perfusion with a physiological fluid, the cOFM perfusate. Tissue reaction due to probe perfusion provides information whether repeated sampling can be performed under comparable conditions.

Tissue reactions such as the formation of a glial scar affect substance exchange between brain tissue and the implanted probe and would therefore strongly limit cOFM usability when sampling interstitial brain fluid. The current study was specifically designed to assess quantitative tissue changes 15 days after probe implantation with a focus on the exchange area at the tip of the cOFM probe. We assessed astrocytes and microglia which are the main components of the glial scar [20]. The quantitative analysis of Iba-1 staining 15 days after cOFM probe implantation revealed only a moderate increase in microglial reaction in the immediate vicinity of the probe tip (<140 µm). In areas more distant from the probe no microglial activation was observed. Also, GFAP staining showed no significant astrocytic reaction along the implantation track 3, 15 and 30 days after cOFM probe implantation. Comparable microdialysis studies have observed a high degree of cell loss, nerve fiber damage, and elevated numbers of astrocytes and microglia up to 300 µm from the probe implantation tracks after 3–14 days [21]–[23]. After a longer implantation time of 30–60 days microdialysis probes even induced the formation of a 2 mm wide glial scar [24], [25]. In the present study implanted microdialysis probes were used as a control for effective stainings and resulted in an increased number of astrocytes and microglia. While histological sections of microdialysis probes include the membrane, the shallow implantation site and the slick surface of the cOFM probe did not allow tissue sectioning with the cOFM probe in situ. Therefore, we checked all explanted cOFM probes with scanning electron microscopy and found no adherent tissue that might have influenced the histological analysis.

In general, cOFM probes caused only very minor tissue reactions and no continuous glial scarring or encapsulation at any time compared to studies using microdialysis probes. A continuous glial scar impairs probe function because it acts as a diffusion barrier between healthy brain tissue and the probe and hampers substance exchange. Methodological investigations have shown that the encapsulation of an implanted probe decreased sensitivity and led to an underestimation of extracellular substance concentrations particularly for larger molecules [26]. Avoiding the formation of a glial scar by using cOFM probes allows unhindered diffusion and emphasizes the potential of cOFM for long-term sampling of various sized molecules under physiological conditions in brain tissue.

One limitation of the present study is the focus on day 15 after cOFM probe implantation when the blood-brain barrier is reestablished and allows unbiased sampling. While the glial scar might not be fully developed at this time, qualitative results indicate a similar minor tissue reaction after 30 days. The qualitative investigation in smaller groups (n = 3) was performed on day 3 and day 30 after cOFM probe implantation in order to show the dynamics of tissue healing. The 30 day group and groups with perfused cOFM probes demonstrate the potential for repeated sampling in experimental setups with a prolonged observation period. The current cOFM sampling system is designed as a preclinical research tool, but technical aspects and used materials are likely to be integrated into a clinical application in the future.

Extensive tissue reaction was avoided by deliberate cOFM probe design. All materials which are in direct contact with brain tissue were selected with the intention to minimize tissue reaction:

The **cOFM guide cannula** is made of FEP (fluorinated ethylene propylene), which is very flexible and has a slick and biologically inert surface. The flexibility of the cOFM guide cannula reduces mechanical stress caused by micro-motions of the brain floating in cerebrospinal fluid while the probe is fixed to the skull [27]. Brain tissue reactions are considerably increased when rigid implants are anchored to the skull compared to the same probe implanted intraparenchymally without fixation to the skull [5]. Increasing the flexibility of an implanted probe decreases thickness of the glial scar and improves neuronal viability in the close vicinity of the probe [28]. The slick surface of the cOFM guide cannula avoids adhesion of immunoreactive cells and proteins that enter the brain when blood vessels are ruptured and meninges are penetrated during implantation [29], [30]. Cell adhesion to rough surfaces or even cell migration into porous structures such as microdialysis membranes [31], [32] were therefore avoided in the cOFM design. The **cOFM** **healing dummy**, a thin steel rod, provides mechanical stability during the implantation process. The tip of the healing dummy is rounded and polished in order to provide a biologically inert surface similar to the guide cannula. The **cOFM perfusate** is adapted to the actual cerebral interstitial fluid in terms of ion content and pH value in order to avoid chemical stress to the brain. The perfusate flow rate was set to 1 µl/min in order to minimize mechanical shear stress to the tissue. The placement of inflow and outflow tubing warms up the perfusate before it reaches the brain tissue and thus avoids temperature stress [33]. Perfusion of the cOFM probe had no significant effect on the astrocytic or microglial reaction, with only a slightly higher cell count in the area up to 140 µm around the probe.

Glial scar formation around the probe is the main limiting factor for long-term implanted sampling systems [34], [35]. We found that the implantation of a cOFM probe did not cause any major tissue reaction during the implantation period of 30 days. Perfusion of the cOFM probes did not alter the histological outcome in the surrounding tissue for the applied stainings.

## Conclusion

cOFM is a new technique for sampling the interstitial brain fluid. Other OFM applications in skin and adipose tissue showed successful sampling of highly lipophilic substances and substances with a high molecular weight. cOFM is intended to provide these benefits for sampling in the brain taking the special conditions in the brain into account. Currently, cOFM probes are mainly used to assess substance transport across the intact blood-brain barrier. Previous studies have shown BBB reestablishment 15 days after cOFM probe implantation. In the present study we did not observe the formation of a continuous glial scar up to 30 days after cOFM probe implantation which would cause probe encapsulation and hamper diffusion between healthy brain tissue and the cOFM probe. Perfusion of the cOFM probes had no significant effect on the tissue around the implantation track so that repeated sampling is feasible. cOFM is a new sampling technique to investigate metabolomic, pharmacodynamic and pharmacokinetic processes and for long-term monitoring in brain tissue.
